# Supplementary material for: Music-evoked reactivation during continuous perception is associated with enhanced subsequent recall of naturalistic events
Source: bioRxiv. 2025 Jul 6:2025.07.05.663273. Preprint. [Version 1] doi: 10.1101/2025.07.05.663273 (PMC12236477; doi:10.1101/2025.07.05.663273)
Supplement: 1 [file NIHPP2025.07.05.663273V1-supplement-1.pdf]

bioRxiv preprint doi: https://doi.org/10.1101/2025.07.05.663273; this version posted July 6, 2025. The copyright holder for this preprint (which was not certified by peer review) is the author/funder, who has granted bioRxiv a license to display the preprint in perpetuity. It is made available under aCC-BY 4.0 International license.

Supplementary Materials

S1: *Auditory Cortex RSA*

To check that participants were encoding the distinctive features of the six songs that we selected in the scanner, we ran a representational similarity analysis (RSA) on music-condition participants to measure whether neural pattern similarity in auditory cortex was greater for same-song comparisons than different-song comparisons. Auditory cortex is well known to play a critical role in representing the acoustic features of music (Peretz et al., 1994; Zatorre et al., 2002; Norman-Haignere et al., 2022), making it a strong candidate for assessing song-level pattern similarity. For this analysis, we computed representational similarity matrices (RSMs) for each individual music participant by correlating their song-specific event patterns with the group average using a leave-one-out approach; these matrices recorded the average pairwise similarity between all 6 songs, measured using Pearson correlation (for same-song comparisons, we only included comparisons between different instances of the same song). Supplementary Figure 1a shows the average of all of the participant-specific RSMs. A one-tailed (on- > off-diagonal) paired samples t-test was conducted to test whether same-song similarity was greater than between-song similarity. We found that same-song pattern similarity was significantly greater than between-song similarity for the selected songs ($t(23) = 13.152$, $p < 0.0001$; Supplementary Figure 1b).

bioRxiv preprint doi: https://doi.org/10.1101/2025.07.05.663273; this version posted July 6, 2025. The copyright holder for this preprint (which was not certified by peer review) is the author/funder, who has granted bioRxiv a license to display the preprint in perpetuity. It is made available under aCC-BY 4.0 International license.

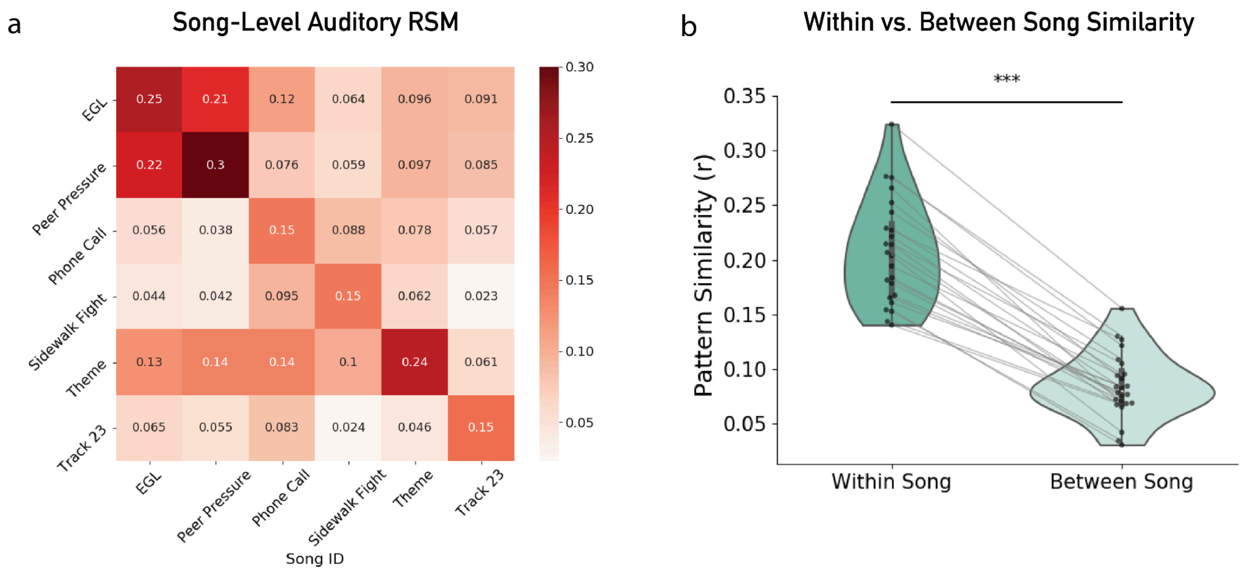

Supplementary Figure 1. Auditory cortex RSA. Panel a displays the RSM for auditory cortex averaged across all participants in the music condition (n=24). Panel b shows the distributions of *r*-values for same-song comparisons and between-song comparisons (each dot represents a participant). T-test results show that within-song similarity is significantly greater than between-song similarity ($t(23) = 13.152$, $p < 0.0001$).

S2: *Remembered vs. Forgotten Reactivation Scores Across All DMN Parcels*

We computed reactivation scores for both remembered and forgotten scenes across all DMN parcels (DMNa, DMNb, and DMNc) within the music and no-music conditions. One-tailed paired samples t-tests were performed between remembered and forgotten scores for each ROI (Supplementary Figure 2). When controlling for multiple comparisons within the music condition, ROIs that survive the threshold criterion ($q \leq 0.1$) include left IPLa-1 ($q = 0.08$), left PMC-4 ($q = 0.03$), and right PMC-2 ($q = 0.08$). No ROIs survived this criterion in the no-music condition.

bioRxiv preprint doi: https://doi.org/10.1101/2025.07.05.663273; this version posted July 6, 2025. The copyright holder for this preprint (which was not certified by peer review) is the author/funder, who has granted bioRxiv a license to display the preprint in perpetuity. It is made available under aCC-BY 4.0 International license.

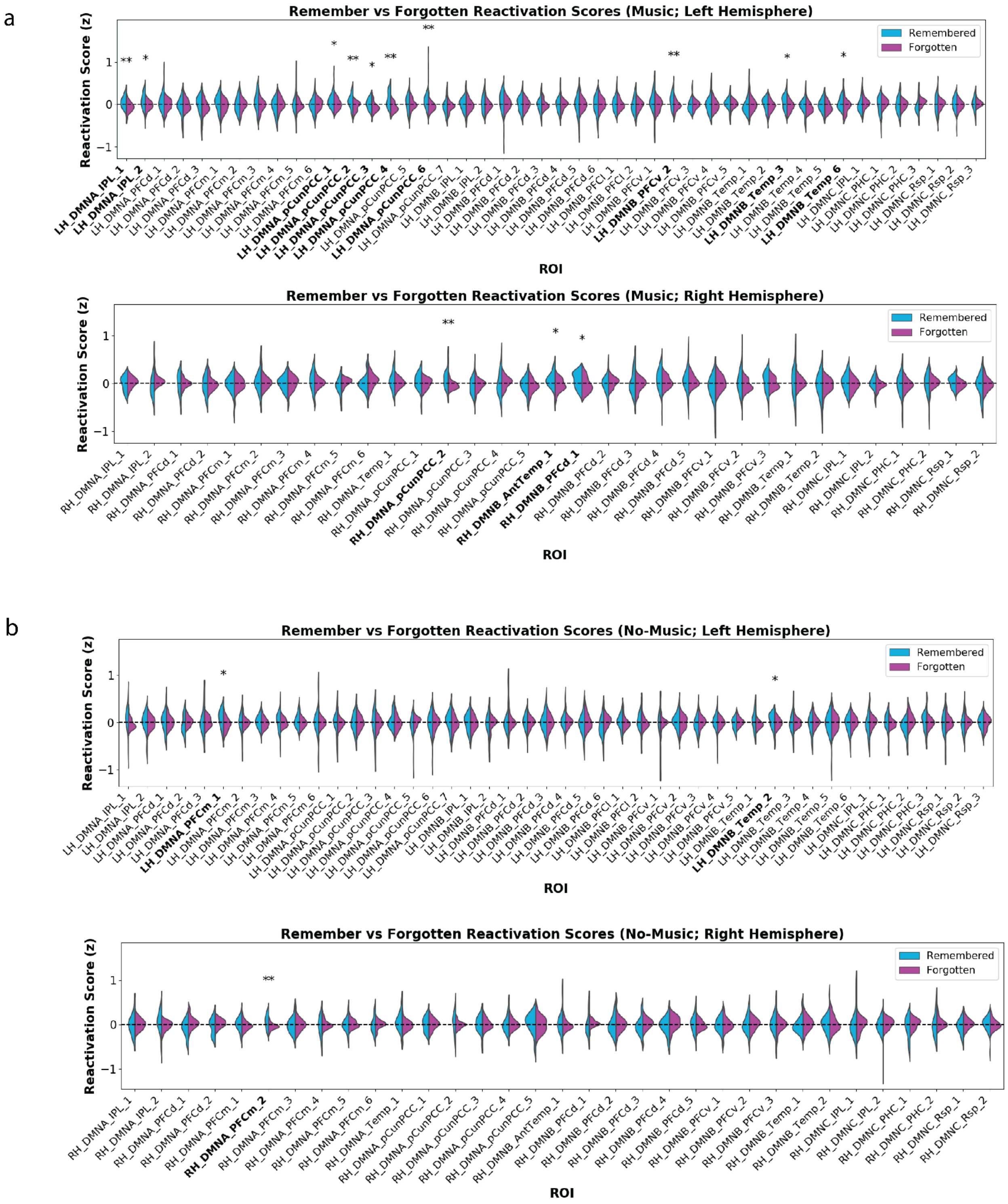

Supplementary Figure 2. Subsequent recall effect for all DMN ROIs. (a) Shows subsequent recall effects for all DMN ROIs (n=79) within the music condition. The top row shows results for the left hemisphere and the bottom row shows results for the right hemisphere. (b) Shows subsequent recall effects for all DMN ROIs in the no-music condition. Significance levels in this plot were not corrected for multiple comparisons (see S2 text for corrected results). Labels of ROIs with uncorrected significance of $p < 0.05$ are bolded. Uncorrected significance is also marked with * for $p < 0.05$ and ** for $p < 0.01$.

bioRxiv preprint doi: https://doi.org/10.1101/2025.07.05.663273; this version posted July 6, 2025. The copyright holder for this preprint (which was not certified by peer review) is the author/funder, who has granted bioRxiv a license to display the preprint in perpetuity. It is made available under aCC-BY 4.0 International license.

S3. *Behavioral Recall Performance*

Participant-level recall performance

We first assessed recall performance at the participant level, computing each participant's mean recall score and then comparing these scores across the music and no-music conditions. This analysis was conducted for three scene categories: *repeated music scenes* (i.e., the scenes included in the neural reactivation analysis)*, all scenes containing music* (regardless of repetition), and *scenes without music*; comparisons were enacted with independent samples, one-tailed (music > no-music) t-tests. Supplementary Figure 3a visualizes these comparisons, where each black dot represents an individual participant's mean recall score.

For *repeated music scenes*, the music group exhibited slightly higher mean recall scores than the no-music group, but this difference was not statistically significant ($t(46) = 0.62$, $p = 0.27$). When considering *all scenes containing music*, we observed a marginally significant trend, with music participants recalling more than no-music participants ($t(46) = 1.47$, $p = 0.074$). Despite not reaching significance, this trend suggests a potential facilitative effect of music on recall. Moreover, it suggests that this facilitative effect may extend to scenes without repeated music (i.e., the presence of music at encoding may boost recall even if that music is not repeated later).

For *scenes without music,* there was no significant difference between the groups ($t(46) = 0.3$, $p = 0.382$), though the music group had a numerically higher recall score.

Scene-Level Recall Performance

Since the same scenes were viewed in the music and no-music conditions, we conducted a scene-level analysis to assess whether differences in free recall performance were present across the two conditions. For each scene, we calculated the proportions of participants recalling it under the music and no-music conditions, and then we computed the difference in these proportions. Supplementary Figure 3b shows the scene-wise differences in recall proportions (*Music – No-Music*) for all scene types. To assess whether these scene-wise differences were reliably positive across scenes (indicating better recall of scenes in the music condition), we ran a one-sample, one-tailed t-test on the difference scores against zero, with degrees of freedom equal to the number of scenes minus one.

We found a significant recall advantage for music in both *repeated music scenes* ($t(66) = 2.28$, $p = 0.013$) and *all scenes containing music* ($t(244) = 6.77$, $p < 0.0001$). However, there was no significant difference for scenes without music ($t(161) = 1.16$, $p = 0.123$).

We also tested whether scene-level recall differences (music minus no-music) were greater for all music scenes compared to scenes without music, and whether differences for repeated music scenes were greater than those for no-music scenes. We found that scene-level recall differences were significantly greater for all music scenes than no-music scenes ($t(405) = 3.18$, $p < 0.001$), but this was not the case when we compared repeated music scenes to no-music scenes ($t(227) = 0.88$, $p = 0.19$).

This scene-level analysis provides a complementary perspective to the participant-level

bioRxiv preprint doi: https://doi.org/10.1101/2025.07.05.663273; this version posted July 6, 2025. The copyright holder for this preprint (which was not certified by peer review) is the author/funder, who has granted bioRxiv a license to display the preprint in perpetuity. It is made available under aCC-BY 4.0 International license.

approach. A limitation of this analysis is that it does not permit generalization to new participants. A benefit of this analysis is that each scene is presented in both the music condition and the no-music condition, permitting use of a paired samples t-test that controls for variability in recall performance across scenes (by contrast, we could not use a paired samples t-test in the participant-level analysis shown in Supplementary Figure 3a, since each participant only appears in one condition).

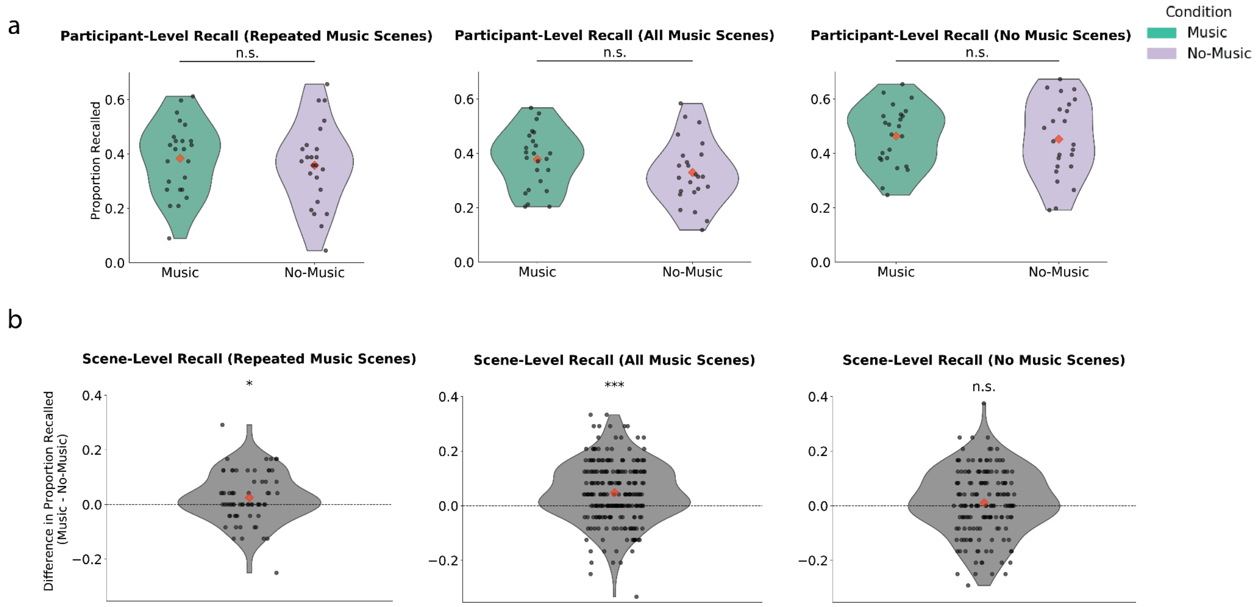

Supplementary Figure 3: Behavioral recall performance between conditions. (a) *Participant-level recall performance by condition for all scene types*. Violin plots show the distribution of proportion recalled scores across participants for the music and no-music conditions within each scene type. Each dot represents an individual participant and red diamonds indicate the group mean. While the music group showed slightly higher mean recall scores for repeated music scenes, the difference did not reach statistical significance ($t(46) = 0.62$, $p = 0.27$). The difference was marginally significant for all scenes containing music ($t(46) = 1.47$, $p = 0.07$); there was no significant difference for scenes that did not contain music ($t(46) = 0.3$, $p = 0.38$). (b) *Scene-level recall performance for all scene types.* Here, each dot represents a scene and its y-axis coordinate plots the difference in proportion recalled (Music - No Music) for that scene. Results are plotted for all scene types. Proportion recalled was significantly greater in the music condition for repeated music scenes ($t(66) = 2.28$, $p = 0.013$) and all music scenes ($t(244) = 6.77$, $p < 0.0001$), but not for scenes that did not contain music ($t(161) = 1.16$, $p = 0.123$). We also found that scene-level differences in proportion recalled were significantly higher for all music scenes than for scenes without music ($t(405) = 3.18$, $p < 0.001$); differences in proportion recalled were numerically higher for repeated music scenes than no-music scenes but these differences were not significant ($t(227) = 0.88$, $p = 0.19$).

S4. *Reactivation Effects (Not Split by Subsequent Memory)*

We tested whether cortical regions exhibited music-evoked reactivation during movie encoding. Analysis steps are the same as those described under *computing reactivation scores* in the *Methods* section, however, reactivation scores were not sorted into bins of remembered or forgotten. Instead significance was tested by evaluating whether subject-level reactivation scores were significantly greater than zero using a one-sample t-test at each ROI. Supplementary Figure 4 shows results when reactivation was tested in a pre-defined set of ROIs (Supplementary Figure 4a) and as a parcel-based searchlight (Supplementary Figure 4b). When performing the analysis

bioRxiv preprint doi: https://doi.org/10.1101/2025.07.05.663273; this version posted July 6, 2025. The copyright holder for this preprint (which was not certified by peer review) is the author/funder, who has granted bioRxiv a license to display the preprint in perpetuity. It is made available under aCC-BY 4.0 International license.

using predefined ROIs, no ROIs showed significant evidence of reactivation. However, when performing the analysis as a parcel-based searchlight, significant reactivation was observed in right angular gyrus ($t(23) = 3.957$, $q < 0.05$) and left parahippocampal cortex ($t(23) = 2.968$, $q < 0.1$).

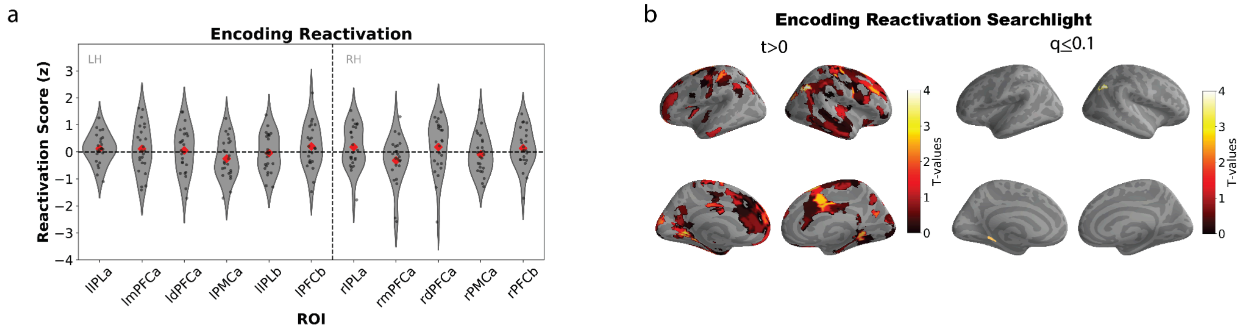

Supplementary Figure 4: Reactivation effects, not split by subsequent memory. (a) First, we tested whether reactivation occurred in regions of the DMN. When testing for this within pre-defined DMN ROIs, we did not observe significant reactivation in these regions. (b) **Left.** Results of full-brain parcel-based searchlight looking for reactivation ($t > 0$). **Right.** Significant reactivation was observed in right angular gyrus and left parahippocampal cortex after correcting for multiple comparisons within the DMN ($q \leq 0.1$).

S5. *Reactivation Predicts Subsequent Recall Searchlight: Statistics for Parcels Passing FDR Correction (Visualized in Figure 1D Right Panel)*

Parcel	T-value (df = 23)	Q-value
Left IPLa-1	3.056	0.08
Left PMCa-2	2.799	0.1
Left PMCa-4	3.925	0.027
Right PMCa-2	3.014	0.08

bioRxiv preprint doi: https://doi.org/10.1101/2025.07.05.663273; this version posted July 6, 2025. The copyright holder for this preprint (which was not certified by peer review) is the author/funder, who has granted bioRxiv a license to display the preprint in perpetuity. It is made available under aCC-BY 4.0 International license.

S6. *Reactivation Predicts Subsequent Recall (Controlling for ISC) Searchlight: Statistics for Parcels Passing FDR Correction (Visualized in Figure 2F Right Panel)*

Parcel	T-value (df = 23)	Q-value
Left IPLa-1	2.631	0.08
Left PMCa-1	2.538	0.09
Left PMCa-2	3.281	0.04
Left PMCa-3	3.103	0.04
Left PMCa-4	3.952	0.03
Left PMCa-6	2.748	0.08
Right PMCa-2	3.121	0.04
Left TEMPb-4	3.122	0.04
